# Changing maternal and child nutrition practices through integrating social and behavior change interventions in community-based self-help and support groups: literature review from Bangladesh, India, and Vietnam

**DOI:** 10.3389/fnut.2024.1464822

**Published:** 2024-11-14

**Authors:** Anumeha Verma, Tuan Nguyen, Appolenarius Purty, Narottam Pradhan, Alomgir Husan, Paul Zambrano, Zeba Mahmud, Sebanti Ghosh, Roger Mathisen, Thomas Forissier

**Affiliations:** ^1^Alive & Thrive, FHI 360, New Delhi, India; ^2^Alive & Thrive, FHI 360 Global Nutrition, Hanoi, Vietnam; ^3^Jeevika, Bihar State Livelihood Promotion Society, Patna, Bihar, India; ^4^Project Concern International, New Delhi, India; ^5^Community Nutrition and Health Activity, CARE, Dhaka, Bangladesh; ^6^Alive & Thrive, FHI 360 Global Nutrition, Manila, Philippines; ^7^Alive & Thrive, FHI 360, Dhaka, Bangladesh; ^8^Alive & Thrive, FHI 360 Global Nutrition, Washington, DC, United States

**Keywords:** breastfeeding, community-based interventions, complementary feeding, maternal nutrition, self-help groups, social and behavior change, support groups

## Abstract

**Introduction:**

Self-help groups (SHGs) and Support Groups (SGs) are increasingly recognized as effective mechanisms for improving maternal and young child nutrition due to their decentralized, community-based structures. While numerous studies have evaluated the outcomes and impact of SHGs and SGs on nutrition practices, there remains a gap in the literature. To address this, we conducted a literature review to examine the role of SHGs and SGs in improving health and nutrition outcomes, focusing on marginalized women, especially pregnant and lactating women (PLW), in India, Bangladesh, and Vietnam, with an emphasis on programs supported by the international non-governmental initiative, Alive & Thrive.

**Methods and materials:**

We conducted a literature review to assess various models, summarizing findings from 34 documents, including research studies, evaluation reports, program materials, strategies, annual reports, work plans, and toolkits. Relevant information from these documents was extracted using predetermined forms.

**Results:**

In India, the models used SHGs with 10–20 women, federated into larger village and district organizations. Bangladesh and Vietnam SGs have similar structures but with local leaders and committees playing key roles. In all three countries, interventions aimed to improve health and nutrition practices through social behavior change (SBC) interventions, including peer-to-peer learning, interpersonal communication, home visits, and community meetings. Outcomes of the interventions showed that SHG members had increased knowledge of breastfeeding, complementary feeding, and improved dietary diversity compared to non-SHG participants. Interventions helped improve infant and young child feeding practices. Common challenges included sustaining the SHGs, ensuring adequate participation, socio-cultural barriers, and logistical difficulties in reaching PLW in remote areas. Limited time for health topics during SHG meetings and the dissolution of older SHGs were also significant issues.

**Conclusion:**

SHG and SG models demonstrate success in improving health and nutrition outcomes but face challenges in scale, sustainability, and participation. Integrating nutrition-focused SBC interventions into SHGs and SGs requires significant capacity building for technical and counseling skills. Ensuring comprehensive coverage and robust quality assessment during community-based rollouts is essential. To sustain these interventions, it is crucial to prevent group dissolution, allow time for maturation, and secure strong stakeholder engagement and political support.

## 1 Introduction

The intergenerational effects and socio-economic costs of undernutrition are well known (1–5). Undernourished women face higher risks of mortality and conditions like anemia, which negatively impact future generations (6, 7). Poor diets, disease, food insecurity, inadequate care, and socio-cultural factors are key causes of undernutrition (8, 9). Women in low and middle-income countries (LMICs), especially in Asia, often face inadequate dietary diversity and low food consumption (10–13). Their diets, especially in low-income settings, are largely based on starches, lacking in nutrient-rich foods (14, 15).

Undernourished children under five face higher risks of disease, lower cognitive ability, and reduced productivity as adults. Children in LMICs in Asia suffer from poor dietary diversity and suboptimal breastfeeding, leading to growth faltering and stunting (16–21). While breastfeeding rates have improved in Bangladesh, India, and Vietnam, early initiation of breastfeeding remains low (22). Various factors like income, education, gender norms, and exposure to nutrition counseling influence breastfeeding practices and overall diet quality for women and children (8, 23–29).

Household behaviors like food distribution, eating preferences, hygiene, education, lack of safe drinking water and health service uptake also contribute to undernutrition (30–35). Social behavior change (SBC) interventions have shown positive results in addressing these issues by influencing behaviors at household, community, and policy levels (31–35). Governments and partners are focusing on strengthening community outreach and capacity building to address these behavioral causes (36–39).

Self-Help Groups (SHGs) and Support Groups (SGs) have emerged as platforms for socio-economic empowerment in low-income communities, especially among women (40–43). There is increasing evidence of their potential to improve health and nutrition outcomes, particularly maternal and infant nutrition (44–49). This paper synthesizes information from models integrating SBC into SHGs and SGs in India, Bangladesh, and Vietnam, focusing on the design, platforms, and challenges.

In India, Jeevika, started by the Bihar government with support from the World Bank in 2006, evolved to include health and nutrition interventions. Rajiv Gandhi Mahila Vikas Pariyojana (RGMVP) in Uttar Pradesh, launched in 2012, also integrated nutrition into its women's empowerment program. Both the programs were initially meant to link SHGs with financial institutions and eventually evolved to include SBC interventions on health and nutrition. In Bangladesh, the Livelihood Improvement of Urban Poor Communities Project (LIUPCP), implemented from 2017 to 2022, organized poor urban communities to address climate resilience and livelihoods along with health and nutrition. Vietnam's Infant Young Child Feeding (IYCF) SG model, developed by Alive & Thrive from 2011 to 2014, focused on reaching ethnic communities in remote areas with maternal and child nutrition information.

While numerous studies have evaluated the outcomes and impact of SHGs and SGs on nutrition practices, there is a lack of comprehensive reviews examining their role in improving health and nutrition outcomes for marginalized women in Asia. To fill in the literature gap, we conducted this literature review to examine the role of SHGs and SGs in improving health and nutrition outcomes, focusing on marginalized women, especially pregnant and lactating women (PLW), in India, Bangladesh, and Vietnam, with an emphasis on programs supported by a non-governmental initiative, Alive & Thrive.

## 2 Methods and materials

### 2.1 Selection of models

The criteria for the selection of models for this review included: (a) implemented in South or Southeast Asia; (b) integrated nutrition services with SHGs or SGs; (c) use of SBC interventions targeting improvement in maternal and child nutrition; (d) involvement of Alive & Thrive either as a technical partner, implementor, or supporting the development partners or governments in any capacity. We have not published any review protocol for this study.

### 2.2 Literature selection

**Figure 1:**
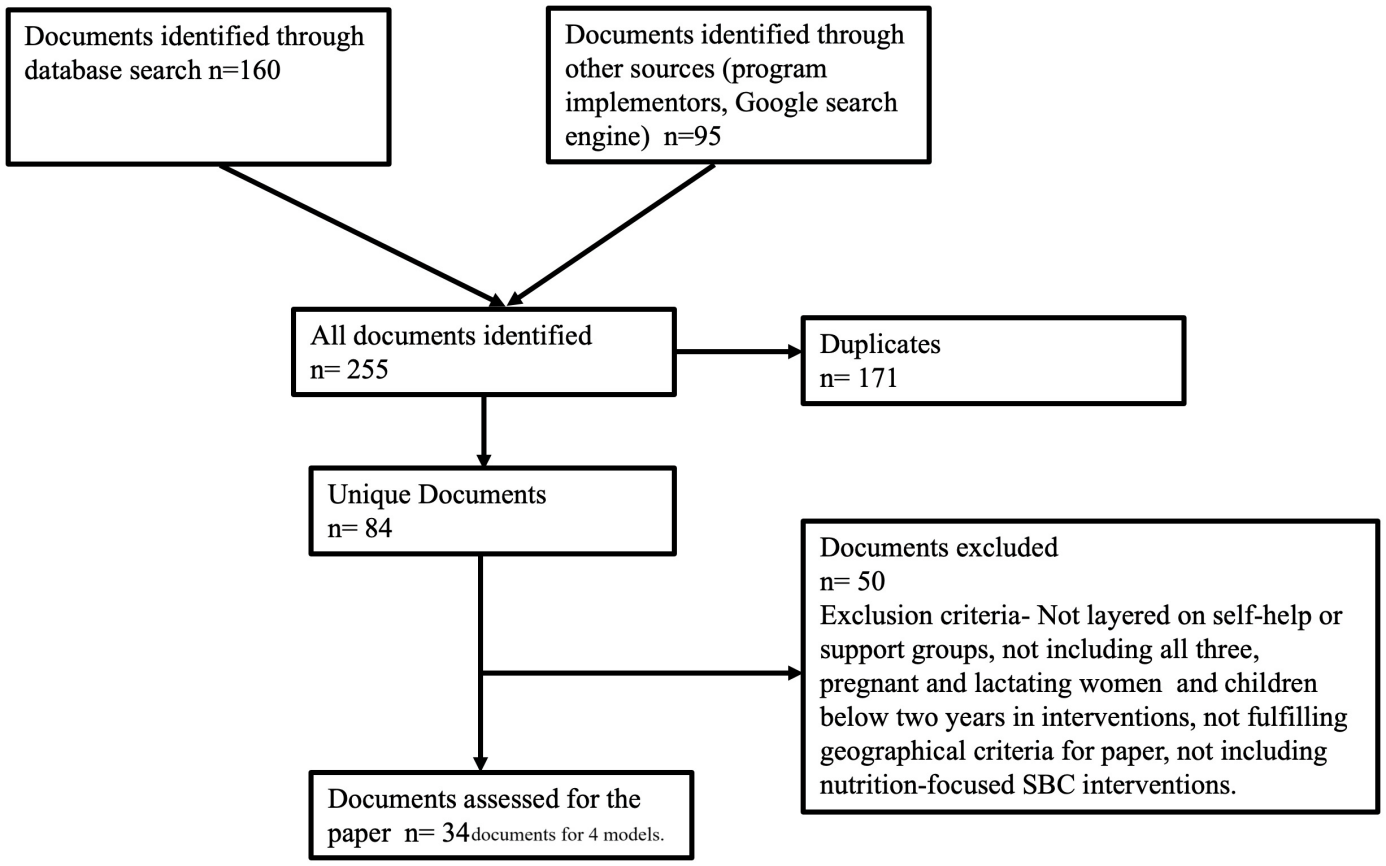
Prisma diagram.

We reviewed the literature to extract information on the selected models and understand how SBC interventions were integrated into the SHGs and SGs. Based on the researchers' language proficiency, we limited our search to English-language documents. The documents included research studies, evaluation reports, program materials, strategies, annual reports, work plans, and toolkits. We placed no restrictions on the publication year. We searched for documents and conducted literature review using three different methods denoted by PRISMA (Figure 1). These included a database search (PUBMED) to select studies on the models using specific key phrases, gathering program materials solicited through program implementors and technical partners, and undertaking a keyword search using Google's search engine to access gray literature and online program materials relevant to the models. We used the same keywords to search through the database and search engine to maintain consistency. We chose the keywords based on the topic, context and models (as defined by the selection criteria). The abstracts obtained through PUBMED were reviewed and selected for further review. The selected literature underwent another round of assessment against the mentioned criteria for a final selection. The documents collected through means other than database search was also assessed for relevance before being admitted for full-length review. The search keywords included following phrases “SHGs in LMICs,” “Health and nutrition integration with SHGs,” Self-Help Groups in India,” “Self-Help Groups in Bangladesh,” “Support Groups in Vietnam,” “Support Groups for IYCF,” “Jeevika,” Rajiv Gandhi Vikas Pariyojana,” “Livelihood improvement for urban poor communities in Bangladesh,” “Support Group Models for Nutrition,” “Nutrition social and behavior change.”

### 2.3 Data items, charting process and synthesis

The whole team discussed the development of key contents for the information extraction forms. The extraction forms include information on methods, platforms, contents, and stakeholders for the Social and Behavior Change Communication (SBC) interventions; program coverage, targeting, and delivery metrics for the training of facilitators who delivered the interventions; framework for integrated program implementation and support; and intervention outcomes.

The lead author extracted information from the selected materials using the defined checklists. Results from the extraction were summarized in tables. The tables, figures, and results were circulated to all authors for review to ensure completeness and accuracy before finalization.

Synthesis of findings were drafted and finalized based on the discussion among all authors.

## 3 Results

Starting with 255 identified documents (Figure 1), the lead author reviewed and excluded 171 due to duplication, and an additional 50 because they did not focus on SHGs or SGs, targeted different groups, did not include SBC interventions, or were from regions outside Asia. The list then was circulated to other co-authors to check for completeness. The documents included for synthesis were research studies in peer-reviewed journals and on other platforms (*n* = 9), evaluations and outcome studies (*n* = 5), and program materials such as program briefs and outcome documents (*n* = 5), strategies (*n* = 4), work plans (*n* = 2), annual reports (*n* = 5), and toolkits (*n* = 4).

### 3.1 Structure and evolution of SHGs and SGs

Table 1 shows that Jeevika's structure includes SHGs with 10–12 members from poor, marginalized households, federated into village organizations and larger clusters. SHGs focus on financial savings, intra-group lending, and linking with banks (50). In 2016, Jeevika reached over seven million households, expanding to more than 10 million (51). Health and nutrition interventions were introduced in 2013, supported by community mobilizers who facilitated SHG meetings and health-related activities. Dedicated nutrition resource persons and Master Resource Persons provided capacity building at the village level, with district-level managers overseeing health, nutrition, and sanitation programs (52).

**Table 1:** Methods and platforms used for social and behavior change communication (SBC) interventions.

	**Jeevika (India)**	**RGMVP (India)**	**LIUCPC (Bangladesh)**	**IYCF SG (Vietnam)**
Duration of the intervention (start and end date)	Pilot: 2012–2016	2011–2018	2018–2024	November 2011–November 2014
	Implementation: 2016–2023			
Methods and platforms used for social and behavior change communication	IPC: SHG meetings, home visits	IPC: SHG meetings, home visits	IPC: PG meetings, home visits, mothers' SGs	IPC: SG meetings with specific topics based on the need of target groups
	Mid-media: wall stickers, posters	Mid-media: rallies, oath-taking, songs in local dialect, wall writing	Mid-media: wall stickers, community events, pamphlets and posters	Mid-media: posters, pamphlets, counseling cards
	Mass-media: a 15-episode drama series aired on YouTube, a digital tool, video shows	Mass-media: video shows	Mass-media: television commercials	Community engagement: involving participants for peer-to-peer support, sharing experiences with each other, food demonstrations, breastfeeding demonstrations
	Community engagement: Cooking and feeding demonstrations, special nutrition drives, involving community members in spreading messages	Community engagement: Quizzes in local dialects, night meetings, special nutrition drives, involving community members in spreading messages	Community engagement: Participation of community members in spreading messages, special drives, developing women and nutrition-friendly business corners, nutrition drives on selected days promoting health and nutrition	Strengthening existing government structures: Working with existing village/commune and primary health centers to strengthen service provision, monitoring and evaluation, and discussions on challenges and course corrections
	Strengthening existing government structures: Community-based events and village health and sanitation day, distribution and production of take-home ration	Strengthening existing government structures: Community-based events and village health and sanitation day	Advocacy: Local governments and organizations providing nutrition services in urban slums, MoHFW	Advocacy: Village heads, local government structures, policymakers at the national level
	Advocacy: With village communities at the local level, local government structures, policymakers	Advocacy: With village communities and village heads, local government structures		
SBC interventions and platforms	Discussions during SHG meetings, line listing of PLW for targeted counseling, targeted home visits for interpersonal counseling, community events, rewards and recognition, support for government instituted CBEs, production and supply of THR, community mobilization for VHSND	Discussions at SHG meetings, counseling through home visits, community, community events, recognition and rewards, nutrition drives and campaigns on maternal and child nutrition, support for government-instituted CBEs	Individual counseling, nutrition education of group members, community mobilization, home visits	Categorizing PLW in three diverse groups, SG meetings, Individual and group counseling, cooking demonstrations, asking each SG member to recall messages from the previous meeting
Key thematic areas for messaging	Antenatal & postnatal care, dietary diversity for PLW, EIBF, EBF, age-appropriate and timely complementary feeding, IFA & Vitamin A supplementation, Integration Child Development Services & other government entitlements for pregnant women and children, kitchen gardens, hygiene practices, handwashing, health risk fund for SHG members	Antenatal & postnatal care, Maternal nutrition during pregnancy & lactation period, EIBF, EBF, debunking myths around breastfeeding, ANC	Antenatal and postnatal care, husband's role during ANC, dietary diversity for PLW, dietary diversity for other primary group members, EIBF, EBF, complementary feeding, dietary diversity for children between 6 and 24 months, not feeding children processed and junk food, handwashing, hygiene, safe drinking water, and sanitation, and support needed by PLW for following recommended practices	Dietary diversity/nutrition adequacy for PLW, EIBF, EBF, breastfeeding techniques, complementary feeding, dietary diversity for children, causes and effects of child malnutrition, significance of breastfeeding, complementary feeding, family & community support required by women for recommended practices
Key actors in nutrition and health service delivery	Nutrition resource persons, MRPs (core block and district staff of Jeevika, community mobilizers, village health sub-committee members, block health, nutrition, and sanitation integrator)	RGVMP staff, community resource persons (Swasthya Sakhi), community leaders from SHGs and federated groups	Socio-Economic and Nutrition Facilitator from LIUPCP, nutrition experts, leaders of primary and federated groups	Community-based workers, commune health staff & provincial and district health staff, members of SGs
Nature of non-SBC interventions	Kitchen garden, livestock rearing, sanitation, livelihood development	Kitchen gardens, sanitation, livelihood development	Conditional cash and food transfer	–
			Nutrition voucher support for adolescent girls	
			Screening of children for malnutrition and referral	
Key departments (convergence) and engagement	Health, ICDS, Lohiya Swacch Bharat Abhiyan agriculture and animal husbandry departments, public distribution system	Health and integrated child development services, agricultural departments	City council, city-level health and nutrition service providers, organizations working on providing sanitation, housing, and other services	Provincial departments of health and reproductive health centers, district health center/commune health center

SBC, Social and Behavior Change; IPC, Interpersonal communication; SG, Support Groups; SHGs, Self Help Groups; MoHFW, Ministry of Health and Family Welfare; PLW, Pregnant and Lactating Women; EIBF, Early Initiation of Breastfeeding; EBF, Exclusive Breastfeeding; IFA, Iron and Folic Acid; ANC, Antenatal Care; MRPs, Master Resource Persons; LIUPCP, Livelihood Improvement of Urban Poor Communities Project; RGMVP, Rajiv Gandhi Mahila Vikas Pariyojana.

RGMVP SHGs also followed a community-centric approach, comprising 10–20 women from marginalized groups. The women were trained for 6 months and then federated into larger organizations. RGMVP focused on socio-economic empowerment through financial inclusion, banking, livelihood, and health services. Nutrition services were introduced through trained mobilizers, with additional focus on maternal and child nutrition (Table 1).

In Bangladesh, the LIUPCP model features three levels of structure. Primary groups of 15–20 members, mostly women, form community development committees, which are further grouped into clusters. These committees focus on nutrition discussions led by designated facilitators (Table 1).

Vietnam's IYCF SG model differs by drawing members from existing village structures. Facilitators, including village health workers and Women's Union members, lead groups focused on breastfeeding, complementary feeding, and community support, targeting pregnant women, mothers, and caregivers in rural areas.

### 3.2 Design of nutrition and health-specific SBC interventions

**Figure 2:**
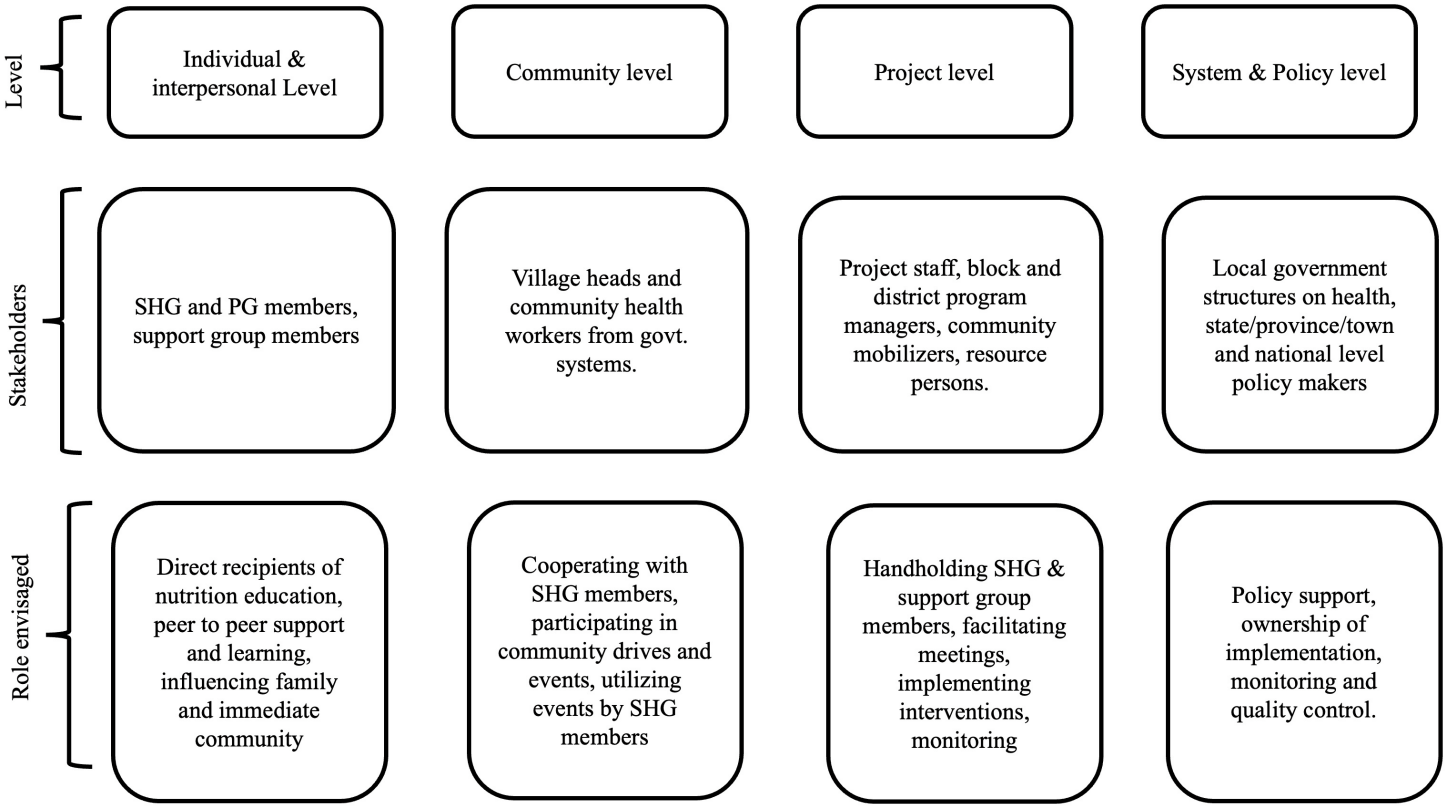
Different levels of stakeholders.

Table 1 also shows that the SBC interventions targeted pregnant women, mothers of children up to 23 months, families, and caregivers. All four models undertook a stakeholder mapping exercise and designed the SBC interventions around the individual, community, program, and policy levels (Figure 2). The three SHG models used a mix of interpersonal communication (IPC), mass media, mid-media, and digital approaches, while the IYCF SG model relied mainly on IPC and on-site demonstrations, using tools like counseling cards and mother-child booklets. SHG contact points included home visits, community events, and nutrition drives, while IYCF SGs focused on village meetings (Table 1).

Coordination with government departments was essential across the SHG and SG models, incorporating SBC, non-SBC nutrition, and nutrition-sensitive interventions. Common methods included storytelling, cooking demonstrations, and peer-to-peer support (Table 1).

Jeevika introduced innovative tools like Samvad Kunji, a digital media tool with QR codes, and food group stickers to monitor dietary diversity. RGMVP used visual maps to help women visualize concerns and plan actions (Table 1). Both programs aimed to shift social norms around maternal nutrition, involving families and communities in the process (53–58).

### 3.3 Coverage and adequacy

Table 2 shows that all models aimed to reach pregnant and lactating women (PLW) and key influencers through SBC interventions. Since not all PLW were SHG members, Jeevika used a two-step approach, identifying PLW within SHG households through members and then reaching them via home visits and community events (53, 54). RGMVP used village maps to track PLW and their needs, combining this with joint home visits. LIUPCP employed similar methods, while the Vietnam IYCF SG model involved village heads to boost community participation and attendance (54, 58, 59).

**Table 2:** Program coverage, targeting, and delivery metrics for the training of facilitators who delivered the interventions by country.

	**Jeevika (India)**	**RGMVP (India)**	**LIUCPC (Bangladesh)**	**IYCF SG (Vietnam)**
**Coverage**				
Number of SHG members and SG facilitators trained	1.02 million (2016 −2023)	Over 10,000 (2011–2017)	139,060 (2018–March 2024)	1,500 (2011–2014)
Total number of PLW who received counseling	13.9 million (between 2016 and 2022)	NA	30,838	30,000
Targeted all relevant program participants (PW, LM with children below 24 months)	Y	Y	Y	Y
Additional direct program participants if any	N	Y	Y	N
**Levels targeted**				
Individual level	Y	Y	Y	Y
Community level	Y	Y	Y	Y
System/policy level	Y	Y	Y	Y
Inclusion non-SBC nutrition interventions	Y	Y	Y	N
**Use of platforms**				
Dedicated training of key actors for delivering nutrition interventions	Y	Y	Y	Y
Two or more platforms used	Y	Y	Y	Y
Three or more interactions with eligible participants from the first trimester of pregnancy till the child became 23 months old	Y	Y	N	Y
Periodic nutrition drives and special campaigns	Y	Y	Y	N
Convergence/coordination with other platforms for service delivery	Y	Y	Y	Y
**Delivery**				
**Type of IEC/SBC materials used**				
Print	Y	Y	Y	Y
Audio-visual	Y	Y	Y	N
Digital	Y	N	N	N
Measures to maintain message consistency (common materials developed; training provided to all concerned members)	Y	Y	Y	Y
BCC materials available to beneficiaries after visits/interventions	Y	Y	Y	Y

SHG, Self Help Group; SG, Support Group; PLW, Pregnant and lactating women; PW, Pregnant women; LM, Lactating mothers; FM, Family members; Y, Yes; N, No; NA, Information not available among reviewed literature; SBC, Social and Behavior Change; IEC, Information, Education and Communication; BCC, Behavior Change Communication.

In 2022, Jeevika targeted 1.82 million mother-child dyads, reaching 45% of PLW in Bihar. LIUPCP in Bangladesh reached over 1.39 million dyads, and Vietnam's IYCF SGs covered 33,000 PLW across nine provinces (53, 54, 58). All models used multiple touchpoints, including weekly and monthly meetings, home visits, and community events to deliver consistent nutrition messages. For example, Jeevika reached each mother 16 times over 6 months, while Vietnam's IYCF model had monthly meetings and occasional community gatherings.

Community mobilization and raising awareness of government nutrition services were central to all models. SHGs played a key role in encouraging participation and promoting nutrition interventions in collaboration with government programs. LIUPCP also organized urban communities to demand services through town federations, while Vietnam's IYCF SG model coordinated efforts with local health systems (61, 62).

### 3.4 Processes and pathways

Table 3 shows that the models integrated SBC interventions into SHGs and SGs due to their strong outreach and mobilization platforms. SHGs target individuals and households in marginalized communities, making them suitable for health and nutrition-focused SBC efforts. In India, SHGs targeted rural poor populations, while in Bangladesh and Vietnam, urban poor and ethnic minorities in remote areas were reached. Prior evidence, local contexts, and formative studies guided the design of interventions, with models like Jeevika using socio-ecological and human-centered design approaches (Table 3).

**Table 3:** Framework for integrated program implementation and support.

	**Jeevika (India)**	**RGMVP (India)**	**LIUCPC (Bangladesh)**	**IYCF SG (Vietnam)**
**Design**				
Rationale for Integration	Nutrition is recognized as a driver for socio-economic and physical wellbeing; common factors between the underlying principles of integrated nutrition interventions and Jeevika; similarities between demographic profiles of target groups; similar overall goals; capacity for outreach; providing agency for women	There are similarities between the demographic profiles of target groups; existing structures and capacity for outreach; similar overall goals; recognition of nutrition as a driver for socio-economic and physical wellbeing; and providing agency for women	Similarities between demographic profiles of target groups; structures for outreach; increased risk of malnutrition due to growing inflation among urban poor; recognition of health and nutrition as a determinant of MPI; lack of health and nutrition services for urban poor; providing agency for women	Providing access to IYCF information for communities in hard-to-reach areas in their own localities; low prevalence of recommended IYCF practices among ethnic minorities; existence of healthcare systems and human resource with some existing knowledge on maternal and child nutrition providing agency to women; contextualizing interventions to local social norms and practices; create an enabling environment for breastfeeding
Evidence	Learnings from Parivartan (change); an initiative by PCI focused on comprehending the dynamics; processes; and effectiveness of BCC interventions in RMNCHN and sanitation interventions in community-based SHGs	Experiences from Society for Elimination of Rural Poverty; Andhra Pradesh on building institutions supported by poor communities	Learnings from the Urban Partnerships for Poverty Reduction project that demonstrated integration of nutrition services with poverty reduction program	Findings from two formative research studies conducted by A&T with support from a technical advisory committee with key stakeholders from UNICEF and the National Institute of Nutrition (NIN) and formative research on trials of improved practices with support from NIN and Ha Noi Medical University and existing studies
	Findings from previous assessment studies in Bihar: Study on IYCF; maternal nutrition; and hygiene practices conducted by A&T in collaboration with PCI; UNICEF; Care; Jeevika Bihar and other existing studies	Findings from formative research and trials of improved maternal nutrition; IYCF; and household sanitation and Hygiene practices in Uttar Pradesh conducted by A&T and other existing studies	Nutrition Survey to assess nutrition status and the prevailing knowledge; practices and nutritional status of children in poor settlements in City Corporations by and Municipalities by UNDP	
	City-Level Nutrition Context Assessment Narayanganj 2019 conducted jointly by A&T and UNDP; Study on understanding opportunities and challenges of delivering MIYCN services in urban areas and MNCH at facilities in Dhaka conducted by A&T and ICDDR; B			
Approach	Socioecological model; human-centered design approach	Socio-ecological model	Socio-ecological model	Behavior change communication combined with supportive supervision and strategic use of data
	Key considerations: evidence; fit with local contexts; gaps and opportunities across various levels for behavior change; existing structures of Jeevika; frequency of interaction; exposure; community participation; and multisectoral approach	Key considerations: local contexts; gaps; existing structures of RGMVP; community participation; and multisectoral approach	Key considerations: evidence; culturally sensitive; local contexts; opportunities across levels; primary and secondary causes of undernutrition; gender sensitivity; promotion of nutrition-focused budgeting; multisectoral approach; and role of local government	Key considerations: access to IYCF information; hard-to-reach areas; cultural sensitivity; localization of interventions; drivers of inadequate IYCF behaviors; social sphere of influence; linkages with existing women unions; village-level structures; and government health systems; the role of local government
**Integration**				
Training	Cadres: Community mobilizers; block HNS nodal HNS; members of village health sub-committee	Cadres: Community health volunteer (one from each SHG) called as Swasthya Sakhi; Meeting Sakhis and members of village organization	Cadres: Socio-economic nutrition facilitator community organizers; local government institutions (municipality and city corporation staff)	Cadres: Community-based workers acting as IYCF facilitators; district trainers
	Materials developed: 20 sessions in 5 modules on health; nutrition; and sanitation covering technical and practical aspects	Materials developed: Modules on newborn and maternal health care; maternal; infant and young child nutrition	Materials developed: Modules on nutrition for field staff along with SBC components; training module on multisectoral urban nutrition to support CLMNCC	Materials developed: 2 training manuals-SG trainer manual and trainee manual
	Approach: cascade	Approach: cascade	Approach: cascade	Approach: cascade
	Mode: Use of classroom interactions; real-life examples; and supplementary materials	Mode: Classrooms; workshops; skits	Modes: classroom teaching	Modes: Classroom interactions; supplementary materials (21 counseling cards)
	Training partners: Jeevika technical support program (JTSP) led by PCI	Training partners: National Institute of Rural Development; Andhra Pradesh; A&T; India; PCI	UNDP's training partners: A&T; Bangladesh	Training partners: save the children
On-ground handholding support	Block HNS nodal person support community mobilizers and SHGs	–	–	Support by village heads
Supervision	MRPs; area and cluster coordinators as well as PCI staff members provided supervision over CMs			IYCF group facilitators were supervised by commune/district health center staff for every meeting; further the District Secretary supervises facilitators every 2 months; A&T staff also provides supervision. All supervisors across levels and staff members used a supportive supervision checklist
Monitoring	Mechanisms: Jeevika decision support system; monthly review meetings by block program managers	–	Mechanisms: Inclusion of MIYCN behavior indicators in LIUPCP project monitoring tool; decentralized review of implementation by community development committees and community development committee clusters	Mechanisms: Multilevel monitoring beginning from data collection by facilitators; proceeding to CHC level; district level; and province level with final reporting to A&T and NIN; Hanoi
	Use: Review of capacity building of MRPs; CMs; orientation of SHGs; mobilization for CBEs and households for kitchen gardens; occurrence of BCC interventions; occurrence of desired behaviors		Use: Review of SENF's performance; the orientation of PGs; the occurrence of planned BCC activities; and the occurrence of desired behavior	Use: Review of occurrence of activities; the occurrence of behaviors; household visits; need for improvement and challenges

RGMVP, Rajiv Gandhi Mahila Vikas Pariyojana; LIUPCP, Livelihood Improvement of Urban Poor Communities Project; IYCF, Infant and Young Child Feeding; BCC, Behavior Change Communication; PCI, Project Concern International; MIYCN, Maternal, Infant and Young Child Nutrition, Maternal Newborn and Child Health; ICDDR, International Center for Diarrheal Disease Research; SHG, Self Help Groups; SG, Support Group; SBC, Social and Behavior Change; CLMNCC, City-Level Multisectoral Nutrition Coordination Committee; JTSP, Jeevika Technical Support Unit; MRPs, Master Resource Persons; CBEs, Community Based Events.

Capacity building was key across all four models, employing a cascade training approach (53–58). Jeevika and RGMVP developed detailed training modules for community mobilizers, nutrition resource persons, and master resource persons (MRPs), combining classroom teaching with participatory methods like role plays and group discussions. By 2017, Jeevika trained 1,500 MRPs, 7,000 nutrition resource persons, and 80,000 community mobilizers, while RGMVP trained over 124,000 community resource persons (Table 3). LIUPCP and Vietnam's IYCF SGs also emphasized training facilitators to lead SBC efforts (62–64).

The models differed in support structures, with Jeevika having a clear ongoing support framework, including post-training assistance and monitoring. Supportive supervision was strong in the Vietnam IYCF SG and present in Jeevika and LIUPCP for nutrition components. Monitoring structures for health and nutrition were clearly defined in most models, except for RGMVP, which tracked improvements during IYCF campaigns. Quality assessment in Jeevika included mobile data collection and feedback mechanisms, while RGMVP tracked knowledge retention and practices in nutrition-focused campaigns (Table 3).

### 3.5 Outcomes, sustainability and scale-up

Table 4 shows that the primary goals of SHGs and SGs were to raise awareness of optimal maternal nutrition, IYCF practices, and government schemes, while improving health and nutrition among mother-child dyads. Outcomes showed that SHG members in Jeevika and LIUPCP had higher knowledge of breastfeeding and complementary feeding than non-members. RGMVP's nutrition campaigns also increased awareness, and Vietnam's IYCF SGs had better outcomes on breastfeeding knowledge. SHG families had higher rates of early breastfeeding initiation and exclusive breastfeeding. Jeevika improved dietary diversity, while RGMVP showed better exclusive breastfeeding rates and increased consumption of Iron Folic Acid (IFA) (60, 64–70).

**Table 4:** Outcomes as cited in various assessment studies.

**Documents reviewed**	**Reported results/findings**
**Jeevika (India)**	
(1) Association of BCC Module Roll-Out in SHG meetings with changes in complementary feeding and dietary diversity among children (6–23 months): Evidence from Jeevika in Rural Bihar, India 300 children (6–23 months), pre- and post-intervention from 60 village organizations Source: PCI (2017)	(1) Adequate dietary diversity for children (eating from at least four food groups out of 7) Pre-intervention: 19% Post-intervention: 49% among SHG members and 28% among non-SHG members. The exposed group had an odds ratio of 3.81 (95% CI: 2.03, 7.15) for consuming a diverse diet compared to the pre-intervention group Higher knowledge of CF: Pre-intervention (48%) and post-intervention (81%), with 91% of the post-intervention respondents being from exposed groups
(2) Study by CARE, 2018 100 JTSP blocks districts in Bihar Vs. Non-JTSP blocks of 11 districts Source: CARE (2018)	(2) Timely initiation of breastfeeding JTSP Blocks: 83%, Non-JTSP blocks: 79% Skin to Skin care JTSP Blocks: 76%, Non-JTSP blocks: 66% Delayed Bathing JTSP Blocks: 62%, Non-JTSP blocks: 55% Initiation of Complementary Feeding JTSP Blocks: 91%, Non-JTSP blocks: 87% MDD JTSP: Blocks: 30%, Non-JTSP blocks: 28% Minimum meal frequency JTSP Blocks: 75%, Non-JTSP blocks: 61%
(3) Endline survey (2023) by Bihar Transformative Development Project (BTDP) 300 Blocks of 32 districts in Bihar Source: BTDP	(3) Children (6–23 months) from targeted SHGs with MDD Baseline: 8%, Midline:23%, Endline: 53% Women from targeted SHGs reporting MDD Baseline: 9%, Midline: 13%, Endline: 54%
(4) Engaging women's groups to improve nutrition: Findings from an evaluation of the JEEViKA multisectoral convergence pilot in Saharsa, Bihar 2246 households from 24 Gram Panchayats of Saharsa district in Bihar (IFPRI's endline survey) Source: IFPRI	(4) 10.3 percentage point (pp) increase in women reporting consuming 5 out of 10 food groups, a 36.3 pp increase in CF practices, an increased number of IFA tablets consumed, and a 2.8 pp increase in the likelihood of women consuming Calcium tablets in treatment arms (all values are in comparison of non-treatment arm) No improvements in the average Body Mass Index (BMI) of women and anthropometric measurements of children, knowledge of services provided by the FLWs (4.7 pp increase over endline comparison mean), knowledge on child feeding (6.6 pp increase over endline comparison mean), knowledge of dietary diversity (4.9 pp increase over endline comparison mean), knowledge on kitchen gardens (2.3 pp increase over endline comparison mean)
**RGMVP (India)**	
(1) Increasing knowledge of home-based maternal and newborn care using self-help groups: Evidence from rural Uttar Pradesh, India SHG women from two administrative blocks of Jhansi district in UP with 25 and 23 GPs, respectively, Baseline 803 participants and endline 470 women Source: Population Council Data	(1) A significant net effect (DID analysis: Net effect 17.4, *p* < 0.05) was observed in knowledge on consuming a minimum of 100 IFA tablets and maternal health in the treatment arm, no significant net effect on knowledge on EIBF, though there were some significant improvements of knowledge on newborn health
(2) CORT Pre and Post survey of one-month educational campaign from a selected block of 41 districts in Uttar Pradesh Participants 419 women baseline (July–August 2018), 506 endline (November 2018). Participants: married women who had given birth to a child within 12 months preceding the survey and/or were currently pregnant. Source: CORT and RGMVP (2018)	(2) 11 pp increase among women at endline on MDD, significant increase (*p* < 0.01) in the knowledge on ANC and services for pregnant women, marginal increase in the number of women consuming IFA tablets
(3) Evaluation of exclusive breastfeeding campaign Participants: 480 (240 SHG + 240 non-SHG) baseline and endline: same as baseline Source: POP Council (2018)	(3) The percentage of mothers practicing exclusive breastfeeding increased from 26.6% to 65.6% (SHG members) and from 29.5% to 58.5% (non-SHG members) Knowledge of exclusive breastfeeding increased among both SHG and non-SHG members, but there was no significant difference between the two groups. SHG members with correct knowledge of early initiation of breastfeeding were more (47.3%−82.4% among SHG members, 75.5% from 47.3% among non-SHG members)
**LIUPCP (Bangladesh)**	
(1) Bi-annual progress report of LIUPCP (October 2022–March 2023) Source: UNDP	(1) 25,796 PLW received nutrition grants, 25,796 Children aged 7–24 months received nutrition 95.2% of PLW grantees consumed protein in the last 7 days. 96.2% of children (7–23 months) grantees consumed protein in the last 24 h (children)
(2) Outcome document of nutrition-specific and nutrition-sensitive interventions and policy advocacy by UNDP Source: UNDP	(2) 81.2% percent of lactating mothers have improved knowledge and skills related to infant and young child feeding practices. 68.9% of targeted (1,000 days) households have improved complementary feeding practices
**IYCF SG (Vietnam)**	
(1) Study by A&T for effectiveness and cost. Participants: 551 mothers of children aged 0–23 months in intervention and 559 in comparison communes from intervention districts in three regions of Vietnam. Source: A&T	(1) Statistically higher (*p* < 0.05) recommended breastfeeding practices (*p* < 0.05) in the intervention communes than in comparison communes (early initiation of breastfeeding (70.6% vs. 58.3%), exclusive breastfeeding under 6 months (62.8% vs. 13.1%), and no bottle feeding (78.0% vs. 60.0%). Mothers in the intervention communes more often fed their children minimum dietary diversity (81.0% vs. 73.0%, *p* = 0.09) and minimum acceptable diet (70.6% vs. 58.3%, *p* = 0.06).
(2) Save the Children Study (2017). 376 samples with 149 intervention households and 227 comparison households from 37 villages in 5 communes. Source: Save the Children	(2) On average, the participants from intervention villages scored 6.0 (out of 10) on the breastfeeding knowledge scale, compared to an average score of 5.3 among the comparison group. On average, mothers from the intervention group scored 4.9 (out of 10) on the complementary feeding knowledge scale, compared to an average score of 4.3 among comparison group mothers. This difference was statistically significant at the 10% level More intervention mothers correctly answered at least half of the questions (79% vs. 67%) and 80 percent of the questions (22% vs. 15%). Appropriate complementary feeding (CF), we found convincing evidence that the project had a positive impact. The intervention group scored significantly higher in CF knowledge compared to the matched comparison group (average score of 4.9 vs. 4.3). A significantly higher proportion of the intervention mothers (58%) was also found to be correctly answering at least half of the questions compared to the mothers from comparison villages (44%)

RGMVP, Rajiv Gandhi Mahila Vikas Pariyojana; LIUPCP, Livelihood Improvement of Urban Poor Communities Project; IYCF, Infant and Young Child Feeding; BCC, Behavior Change Communication; PCI, Project Concern International; MIYCN, Maternal, Infant and Young Child Nutrition, Maternal Newborn and Child Health; ICDDR, International Center for Diarrheal Disease Research; SHG, Self Help Groups; SG, Support Group; SBC, Social and Behavior Change; CLMNCC, City-Level Multisectoral Nutrition Coordination Committee; JTSP, Jeevika Technical Support Unit; MRPs, Master Resource Persons; CBEs, Community Based Events.

Sustainability and scale-up showed mixed results. Jeevika expanded from 101 blocks to 300 blocks in Bihar, supported by the state government and the World Bank (70). It contributed to India's National Rural Livelihood Mission (NRLM), integrating food, nutrition, and health initiatives. RGMVP, active in 49 districts at its peak, saw a decline after donor support ended in 2018, though learnings informed future models (Table 4).

LIUPCP in Bangladesh, ending in 2024, proposed multisectoral coordination for sustained nutrition efforts, with some cities already operating independently. Vietnam's IYCF SG model scaled up successfully, covering 267 villages in nine provinces, supported by local government and partners like Save the Children and World Vision (Table 4). The model adapted to local investments for long-term sustainability (71–73).

### 3.6 Challenges

SHGs faced critical challenges including adequacy, sustainability, and quality assessment. Jeevika, RGMVP, and LIUPCP, initially focused on livelihood, poverty reduction, and financial inclusion, were not designed for health and nutrition interventions, requiring significant capacity building and new components, increasing the workload for community mobilizers.

SHGs had gaps in time allocation and meeting frequency. Jeevika's groups met weekly for about 30 min on health topics aside from training, while RGMVP had monthly meetings with similar time for health discussions. Meeting frequency varied, especially during harvest season. In Vietnam, IYCF SG meetings occurred monthly for PLW and bimonthly for others, with messages reviewed at subsequent meetings.

Sustaining SHGs proved challenging, with dissolutions reported for Jeevika and RGMVP, impacting HNS component implementation. Low meeting participation also hindered SBC interventions. Most women in Jeevika, RGMVP, and LIUPCP were not of reproductive age, leading to reliance on home visits and community events to reach PLW, which saw low attendance in some cases. Jeevika's assessment revealed some CMs lacked necessary skills, leading to refresher training.

The Vietnam IYCF SG model faces socio-cultural and economic barriers, with traditional practices and limited resources affecting adherence to recommended feeding practices. Remote areas struggle with healthcare access, and post-support from Alive & Thrive, reduced funding led to decreased meeting frequency. While a sustainability plan is in place, not all communities can fund activities beyond national program support.

### 3.7 Summary of findings

SHGs and SGs are recognized as effective community-based models for improving maternal and child nutrition. This literature review, focusing on India, Bangladesh, and Vietnam, explores their role in enhancing health and nutrition outcomes, particularly for PLW from marginalized communities. Drawing from 34 documents, the review highlights that SHGs in these countries use decentralized, peer-driven approaches to deliver social behavior change interventions like peer learning, interpersonal communication, and community events. These interventions have improved knowledge of breastfeeding, complementary feeding, and dietary diversity among SHG members. However, challenges such as sustaining group participation, overcoming socio-cultural barriers, and logistical difficulties remain significant.

Sustainability and fidelity issues arose from low participation, irregular meetings, and capacity gaps. Economic barriers, traditional practices, and reduced support also hindered activity sustainability, despite plans in place.

## 4 Discussion

Our synthesis shows that while integrating SBC interventions for MIYCN into SHGs and SGs produces encouraging outcomes, key lessons must be learned about designing and implementing these interventions, especially with regard to long-term sustainability and scalability. With the growing focus of global funding bodies and national governments on community-led and localized development, SHGs and SGs gain further significance as platforms embedded within communities (74). Recently, development partners and funding organizations have provided evidence supporting demand-driven capacity building, institutionalizing feedback and accountability within communities, and making monitoring, learning, and evaluation more participatory for successful community-led development (75, 76).

The integration processes must feature intensive capacity building for SHGs, SG meeting facilitators, and community mobilizers. Earlier studies have also highlighted the need for capacity building in community-based interventions to empower communities and place their voices at the center of solving the challenges that affect them (77–79). Our synthesis showed that the models focused on developing training materials that combine technical information with soft skills to maintain consistency in delivering training and orienting key actors to build their knowledge and counseling skills. Implementing agencies collaborated with technical partners, which significantly aided this process.

A significant challenge in implementing MIYCN-focused interventions is that although SHGs provide a suitable platform, their reach is not always direct. While PLW are members of SHG households, they are not necessarily direct members of SHGs themselves. Therefore, identifying ways to reach PLW and their influencers during the design phase is essential. Strategies such as listing identified PLW, conducting home visits, organizing open-to-all community events, and hosting nutrition drives or campaign-like events were some of the pathways used by the reviewed models to ensure coverage of all target groups. A previous systematic review on behavioral change interventions to improve maternal and child nutrition in sub-Saharan Africa also shows positive impacts of interventions based on behavior change theory, counseling, and communication (79). These interventions improved infant and child nutrition outcomes by reducing wasting, underweight, and stunting, and enhancing dietary diversity and total food consumption, as well as maternal psychological outcomes. Additionally, this study shows that interventions incorporating the Behavior Change Wheel functions (incentivization, persuasion, and environmental restructuring) were most effective (79).

Beyond reach, the time allocated for nutrition discussions in SHGs and the frequency of meetings are equally crucial, as SHG members are expected to amplify nutrition messages beyond the group. There are encouraging examples of intensity when we consider the frequency of interactions with PLW. These groups, particularly those that followed a layered approach with multiple interventions, enabled multiple contact points with target groups. Studies from various countries have emphasized the benefits of multiple contact points for improving MIYCN outcomes (79, 80), which was made possible through SHGs. SGs dedicated to PLW do not face this challenge. However, SG models must work with influencers to ensure attendance at meetings and secure buy-in from existing health structures to guarantee the availability of facilitators and government ownership.

Our review also showed that engaging influencers at the policy level is essential to position maternal and child nutrition as critical for both health and economic productivity outcomes and to garner support for community institutions. This finding aligns with previous studies that deem advocacy at the policy level crucial for the success of health and nutrition interventions (81–83). Political will and policy-level support played a significant role in sustaining the Jeevika model. The scale-up and successful adaptation of CLMNCC under the LIUPCP program in Bangladesh, as well as the implementation of the Vietnam IYCF SG, demonstrated the substantial role of policy advocacy in ensuring effective program implementation, monitoring, and review through existing government systems.

This study has limitations. This scoping review focuses on four known Alive &Thrive programs and the processes and outcomes reported in the selected documents. Therefore, this review might not capture information from other programs and interventions, making it an internal organizational review, which could theoretically cause bias toward positive outcomes. Although we were not able to address such biases, to our knowledge, there are no other similar interventions at the project sites, and the majority of documents used for this study were peer-reviewed publications and published reports. A previous study indicates that behavior change communication might not be sufficient (79). Since we are not able to evaluate background information beyond the intervention, the effect could be the result of other interventions in the same community, such as food supplementation, cash transfers, mass communications, or general improvements in socioeconomic status (79). Further research is needed to better understand the influence of different aspects of these models and to identify which attributes are most associated with impact.

Additionally, we acknowledge that this was a scoping review rather than a systematic review, and we could not use search engines other than PubMed. Embase, Web of Science, and Scopus which are subscription-based databases that were not permitted by the donor due to their associated costs. Our search in the Cochrane Library did not yield any relevant literature reviews. Also, due to resource constraints, we could only arrange for one author within our organization to perform article screening and data extraction, and no formal software or tools were used to manage the process or evaluate the quality of documents. Given that the findings were reviewed by authors who have worked with these programs from the beginning, we anticipate that key literature and information have been captured.

In conclusion, SHG-based models have demonstrated success in improving health and nutrition outcomes but face challenges related to scale, sustainability, and participation. To address these challenges, it is essential to strengthen these models by maintaining rigorous and intense implementation, providing high-quality capacity building, conducting regular assessments, securing policy support, and ensuring sustained political commitment. Additionally, SHG models should be closely monitored and documented to bolster advocacy, generate political will, and foster ownership. The findings from this study can be utilized by policymakers, project managers, scholars, health workers, and frontline workers in designing, planning, implementing, and evaluating relevant intervention models in low-resource settings of lower-middle-income countries.

## Data Availability

The original contributions presented in the study are included in the article/supplementary material, further inquiries can be directed to the corresponding author.

## References

[1] Jain S Ahsan S Robb Z Crowley B Walters D. The cost of inaction: a global tool to inform nutrition policy and investment decisions on global nutrition targets. Health Policy Plan. (2024) 39:819–30. 10.1093/heapol/czae056 39016340 PMC11384108

[2] Caleyachetty R Kumar NS Bekele H Manaseki-Holland S. Socioeconomic and urban-rural inequalities in the population-level double burden of child malnutrition in the East and Southern African Region. PLOS Glob Public Health. (2023) 3:e0000397. 10.1371/journal.pgph.0000397 37097991 PMC10128925

[3] Govindaraj R Premkumar A Sivasankar V. Prevalence and assessment of child malnutrition in South Asia. MGM J Med Sci. (2024) 10:685–90. 10.4103/mgmj.mgmj_246_23

[4] Haddad L Cameron L Barnett I. The double burden of malnutrition in SE Asia and the Pacific: priorities, policies and politics. Health Policy Plan. (2015) 30:1193–206. 10.1093/heapol/czu110 25324529

[5] The World Bank. The World Bank and Nutrition. Available at: https://www.worldbank.org/en/topic/nutrition/overview#1 (accessed September 18, 2024).

[6] Shenoy S Sharma P Rao A Aparna N Adenikinju D Iloegbu C . Evidence-based interventions to reduce maternal malnutrition in low and middle-income countries: a systematic review. Front Health Serv. (2023) 3:1155928. 10.3389/frhs.2023.1155928 37954061 PMC10634505

[7] United Nations Children's Fund (UNICEF). Undernourished and Overlooked: A Global Nutrition Crisis in Adolescent Girls and Women. UNICEF Child Nutrition Report Series, 2022. New York, NY: UNICEF (2023).

[8] Nguyen PH Kachwaha S Tran LM Sanghvi T Ghosh S Kulkarni B . Maternal diets in India: gaps, barriers, and opportunities *Nutrients*. (2021) 13:3534. 10.3390/nu13103534 34684535 PMC8540854

[9] Infant and Young Child Feeding: Model Chapter for Textbooks for Medical Students and Allied Health Professionals. Geneva: World Health Organization (2009). Available at: https://www.ncbi.nlm.nih.gov/books/NBK148967/ (accessed January 3, 2024). 23905206

[10] Islam MH Nayan MM Jubayer A Amin MR. A review of the dietary diversity and micronutrient adequacy among the women of reproductive age in low- and middle-income countries. Food Sci Nutr. (2023) 12:1367–79. 10.1002/fsn3.3855 38455218 PMC10916566

[11] Shaun MMA Nizum MWR Shuvo MA Fayeza F Faruk MO Alam MF . Determinants of minimum dietary diversity of lactating mothers in the rural northern region of Bangladesh: a community-based cross-sectional study. Heliyon. (2023) 9:e12776. 10.1016/j.heliyon.2022.e12776 36632115 PMC9826838

[12] Drummond E Watson F Blankenship J. UNICEF East Asia and the Pacific Regional Office, Nutrition Section, UNICEF, Bill and Melinda Gates Foundation, et al. Southeast Asia Regional Report on Maternal Nutrition and Complementary Feeding. Available at: https://www.unicef.org/eap/media/9466/file/Maternal%20Nutrition%20and%20Complementary%20Feeding%20Regional%20Report.pdf (accessed January 3, 2024).

[13] FHI Solutions. Closing the Gender Nutrition Gap: An Action Agenda for women and girls. (2023). Available at: https://gendernutritiongap.org/wp-content/uploads/2023/07/The-Gender-Nutrition-Gap-an-Action-Agenda-for-women-and-girls.-July-2023.-1.pdf (accessed February 5, 2024).

[14] Shumayla S Irfan EM Kathuria N Rathi SK Srivastava S Mehra S . Minimum dietary diversity and associated factors among lactating mothers in Haryana, India: a community based cross-sectional study. BMC Pediatr. (2022) 22:525. 10.1186/s12887-022-03588-5 36057585 PMC9440519

[15] TCI (Tata–Cornell Institute). Food, Agriculture, and Nutrition in South Asia. Ithaca, NY: TCI. (2023). Available at: https://tci.cornell.edu/wp-content/uploads/2024/01/FAN-South-Asia-2023.pdf (accessed January 5, 2024).

[16] Laelago Ersado T. Causes of malnutrition. In:Saeed F Ahmed A Afzaal A, editors. Combating Malnutrition through Sustainable Approaches. London: IntechOpen. (2023). 10.5772/intechopen.104458

[17] Mohammad A Khan A. Child malnutrition and mortality in South Asia: a comparative analysis. Eur Econ Lett. (2024) 14:1008–18. 10.1177/097152310701400110

[18] Kurniasih D Al Zaharo R Nursamtari R Januarti M. Stunting prevention in low and middle income countries. In:Adurokhim, editor. Published Conference Proceedings of International Conference on Scientific Studies. (2023). Jave, Indonesia. Available at: https://scientists.internationaljournallabs.com/index.php/sc/article/view/6/6 (accessed September 8, 2024).

[19] Lassi ZS Irfan O Hadi R Das JK Bhutta ZA. PROTOCOL: effects of interventions for infant and young child feeding (IYCF) promotion on optimal IYCF practices, nutrition, growth, and health in low- and middle-income countries: a systematic review. Campbell Syst Rev. (2018) 14:1–26. 10.1002/CL2.189 37131389 PMC8427994

[20] Tran LM Nguyen PH Young MF Martorell R Ramakrishnan U. The relationships between optimal infant feeding practices and child development and attained height at age 2 years and 6-7 years. Matern Child Nutr. (2024) 20:e13631. 10.1111/mcn.13631 38450914 PMC11168365

[21] Gatica-Domínguez G Neves PAR Barros AJD Victora CG. Complementary feeding practices in 80 low- and middle-income countries: prevalence of and socioeconomic inequalities in dietary diversity, meal frequency, and dietary adequacy. J Nutr. (2021) 151:1956–64. 10.1093/jn/nxab088 33847352 PMC8245881

[22] Global Breastfeeding Collective. Global Scorecard. [Database] Availabel at: https://www.globalbreastfeedingcollective.org/about-collective (accessed December 15, 2023).

[23] Rahman MA Kundu S Rashid HO Tohan MM Islam MA. Socio-economic inequalities in and factors associated with minimum dietary diversity among children aged 6–23 months in South Asia: a decomposition analysis. BMJ Open. (2023) 13:e072775. 10.1136/bmjopen-2023-072775 38128933 PMC10749007

[24] Lakhanpaul M Roy S Benton L Lall M Khanna R. Vijay VK, et al. Why India is struggling to feed their young children? A qualitative analysis for tribal communities. BMJ Open. (2022) 12:e051558. 10.1136/bmjopen-2021-051558 35902199 PMC9341212

[25] Nguyen TT Nguyen PH Hajeebhoy N Nguyen HV Frongillo EA. Infant and young child feeding practices differ by ethnicity of Vietnamese mothers. BMC Pregnancy Childbirth. (2016) 16:214. 10.1186/s12884-016-0995-8 27502920 PMC4977888

[26] Gokhale D Rao S. Socio-economic and socio-demographic determinants of diet diversity among rural pregnant women from Pune, India. BMC Nutr. (2022) 8:54. 10.1186/s40795-022-00547-2 35787284 PMC9254638

[27] Nguyen PH Kachwaha S Avula R Young M Tran LM Ghosh S . Maternal nutrition practices in Uttar Pradesh, India: role of key influential demand and supply factors. Matern Child Nutr. (2019) 15:e12839. 10.1111/mcn.12839 31066195 PMC6852235

[28] Haque S Salman M Hossain MS Saha SM Farquhar S Hoque MN . Factors associated with child and maternal dietary diversity in the urban areas of Bangladesh. Food Sci Nutr. (2023) 12:419–29. 10.1002/fsn3.3755 38268877 PMC10804084

[29] Banu B Haque S Shammi SA Hossain MA. Maternal nutrition knowledge and determinants of the child nutritional status in the northern region of Bangladesh. Bangladesh J Multidiscip Sci Res. (2023) 7:11–21. 10.46281/bjmsr.v7i1.2018

[30] Nguyen PH Headey D Frongillo EA Tran LM Rawat R Ruel MT . Changes in underlying determinants explain rapid increases in child linear growth in alive and thrive study areas between 2010 and 2014 in Bangladesh and Vietnam. J Nutr. (2017) 147:462–9. 10.3945/jn.116.243949 28122930 PMC5320405

[31] Simwanza NR Kalungwe M Karonga T Mtambo CMM Ekpenyong MS Nyashanu M . Exploring the risk factors of child malnutrition in Sub-Sahara Africa: a scoping review. Nutr Health. (2023) 29:61–9. 10.1177/02601060221090699 35369816

[32] Chowdhury MRK Rahman MS Billah B Rashid M Almroth M Kader M . Prevalence and factors associated with severe undernutrition among under-5 children in Bangladesh, Pakistan, and Nepal: a comparative study using multilevel analysis. Sci Rep. (2023) 13:10183. 10.1038/s41598-023-36048-w 37349482 PMC10287716

[33] Workicho A Biadgilign S Kershaw M Gizaw R Stickland J Assefa W . Social and behaviour change communication to improve child feeding practices in Ethiopia: *Matern Child Nutr*. (2021) 17:e13231. 10.1111/mcn.13231 34132054 PMC8476421

[34] Kennedy E Stickland J Kershaw M Biadgilign S. Impact of social and behavior change communication in nutrition specific interventions on selected indicators of nutritional status. J Hum Nutr. (2018) 2:34–46. 10.36959/487/280

[35] Sanghvi T Haque R Roy S Afsana K Seidel R Islam S . Achieving behaviour change at scale: Alive and Thrive's infant and young child feeding programme in Bangladesh. Matern Child Nutr. (2016) 12:141–54. 10.1111/mcn.12277 27187912 PMC6680185

[36] Flax VL Bose S Escobar-DeMarco J Frongillo EA. Changing maternal, infant and young child nutrition practices through social and behaviour change interventions implemented at scale: lessons learned from Alive and Thrive. Matern Child Nutr. (2023) e13559. 10.1111/mcn.13559 37735818 PMC11956063

[37] Litvin K Grandner GW Phillips E Sherburne L Craig HC. Kieu Anh Phan, et al. How do social and behavioral change interventions respond to social norms to improve women's diets in low- and middle-income countries? A scoping review. Curr Dev Nutr. (2024) 8:103772–2. 10.1016/j.cdnut.2024.103772 38948109 PMC11214384

[38] Metcalfe-Hough V Fenton W Saez P Spencer A. The Grand Bargain in 2021: an independent review. HPG commissioned report. London: ODI. (2021). Available at: https://interagencystandingcommittee.org/sites/default/files/migrated/2022-06/Grand%20Bargain%20Annual%20Independent%20Report%202022.pdf

[39] USAID. Localization at USAID: the vision and approach. (2022). Available at: https://www.usaid.gov/sites/default/files/2022-12/USAIDs_Localization_Vision-508.pdf (accessed March 2, 2024).

[40] Ghosh S Mahapatra MS Tandon N Tandon D. Achieving sustainable development goal of women empowerment: a study among self-help groups in India. FIIB Bus Rev. (2024) 13:477–91. 10.1177/2319714523116907437079532

[41] WHO. Essential nutrition actions: Improving maternal, newborn, infant and young child health and nutrition. Available at: https://iris.who.int/bitstream/handle/10665/84409/9789241505550_eng.pdf?sequence=1World Bank (accessed April 30, 2024). 25473713

[42] World Bank. World Bank Support to Reducing Child Undernutrition. Independent Evaluation Group. Washington, DC: World Bank (2021).

[43] Nichols C. Self-help groups as platforms for development: the role of social capital. World Dev. (2021) 146:105575. 10.1016/j.worlddev.2021.105575 34602707 PMC8350316

[44] Bhanot A Sethi V Murira Z Singh KD Ghosh S Forissier T . Right message, right medium, right time: powering counseling to improve maternal, infant, and young child nutrition in South Asia. Front Nutr. (2023) 10:1205620. 10.3389/fnut.2023.1205620 37743925 PMC10512175

[45] Desai S Misra M Das A Singh RJ Sehgal M Gram L . Community interventions with women's groups to improve women's and children's health in India: a mixed-methods systematic review of effects, enablers and barriers. BMJ Glob Health. (2020) 5:e003304. 10.1136/bmjgh-2020-003304 33328199 PMC7745316

[46] Saggurti N Atmavilas Y Porwal A Schooley J Das R Kande N . Effect of health intervention integration within women's self-help groups on collectivization and healthy practices around reproductive, maternal, neonatal and child health in rural India. PLoS ONE. (2018) 13:e0202562. 10.1371/journal.pone.0202562 30138397 PMC6107172

[47] Sethi V Bhanot A Bhalla S Bhattacharjee S Daniel A Sharma DM . Partnering with women collectives for delivering essential women's nutrition interventions in tribal areas of eastern India: a scoping study. J Health Popul Nutr. (2017) 36:20. 10.1186/s41043-017-0099-8 28532433 PMC5441055

[48] Scott S Gupta S Kumar N Raghunathan K Thai G Quisumbing A . A women's group-based nutrition behavior change intervention in India has limited impacts amidst implementation barriers and a concurrent national behavior change Campaign. Curr Dev Nutr. (2021) 5(Supplement 2):179. 10.1093/cdn/nzab035_087

[49] Husain Z Dutta M. Impact of Self Help Group membership on the adoption of child nutritional practices: evidence from JEEViKA's health and nutrition strategy programme in Bihar, India. J Int Dev. (2023) 35:781–99. 10.1002/jid.3703

[50] Pradhan MR Unisa S Rawat R Surabhi S Saraswat A Reshmi SR . Women empowerment through involvement in community-based health and nutrition interventions: evidence from a qualitative study in India. PLoS ONE. (2023) 18:e0284521. 10.1371/journal.pone.0284521 37079532 PMC10118087

[51] UNICEF ENN Project Concern International India. Delivering for Nutrition in South Asia: equity and inclusion (2023). Available at: https://www.ennonline.net/sites/default/files/2024-01/delivering_for_nutrition_in_south_asia_equity_and_inclusion.pdf (accessed March 5, 2024).

[52] Hazra A Das A Ahmad J Singh S Chaudhuri I Purty A . Matching intent with intensity: implementation research on the intensity of health and nutrition programs with women's self-help groups in India. Glob Health Sci Pract. (2022) 10:e2100383. 10.9745/GHSP-D-21-00383 35487547 PMC9053147

[53] Hora G Krishna P Singh RK Ranjan A. Lessons from a decade of rural transformation in Bihar. in JEEViKA Learning Note Series. (2019). Available at: https://documents1.worldbank.org/curated/en/298391515516507115/122290272_20180012032255/additional/122548-WP-P090764-PUBLIC-India-BRLP-Booklet-p.pdf (accessed December 1, 2023).

[54] Mozumdar A Khan M Mondal SK Mohanan P. Increasing knowledge of home based maternal and newborn care using self-help groups: evidence from rural Uttar Pradesh, India. Sex Reprod Healthc. (2018) 18:1–9. 10.1016/j.srhc.2018.08.003 30420079

[55] Hazra A Atmavilas Y Hay K Saggurti N Verma RK Ahmad J . Effects of health behaviour change intervention through women's self-help groups on maternal and newborn health practices and related inequalities in rural india: a quasi-experimental study. EClinicalMedicine. (2019) 18:100198. 10.1016/j.eclinm.2019.10.011 31993574 PMC6978187

[56] Jeevika. Transforming Lives: Social and Behavior Change for Child Health and Nutrition in Bihar. New Delhi: PCI (2024).

[57] UNDP Bangladesh and Alive & Thrive. Nutrition Social and Behavior Change Strategy for the Livelihoods Improvement of Urban Poor Communities Project (LIUPCP). Dhaka: UNDP Bangladesh [Report] (2021).

[58] Alive and Thrive. Overview of the Alive and Thrive Infant and Young Child Feeding Community-based SG model in Viet Nam. (2013). Available at: https://www.aliveandthrive.org/sites/default/files/attachments/IYCF-SG-Four-Pager-English-Sept-2013.pdf (accessed January 12, 2024).

[59] Rajiv Gandhi Charitable Trust. Annual Report 2017-2018. (2018). Available at: https://www.rgct.in/pdfs/annual_report_2017_18.pdf (accessed February 5, 2024).

[60] National Urban Poverty Reduction Programme (NUPRP). National Urban Poverty Reduction Programme (NUPRP) Bi-Annual Progress Report October 2022-March 2023 [Report] (2024). Available at: https://erc.undp.org/evaluation/documents/download/23704

[61] Kabir AFMI Livelihoods Livelihoods Improvement of Urban Poor Communities Project (LIUPCP). Outcome Documentation of Nutrition Sensitive and Specific Interventions, and Policy Advocacy. [Report]. Dhaka (2024).

[62] Nguyen TT Hajeebhoy N Li J Do CT Mathisen R Frongillo EA . Community support model on breastfeeding and complementary feeding practices in remote areas in Vietnam: implementation, cost, and effectiveness. Int J Equity Health. (2021) 20:121. 10.1186/s12939-021-01451-0 34001154 PMC8127246

[63] Irani L Schooley J Supriya Chaudhuri I. Layering of a health, nutrition and sanitation programme onto microfinance-oriented self-help groups in rural India: results from a process evaluation. BMC Public Health. (2021) 21:2131. 10.1186/s12889-021-12049-0 34801003 PMC8605516

[64] Mondal S Joe W Akhauri S Thakur P Kumar A Pradhan N . Association of BCC module roll-out in SHG meetings with changes in complementary feeding and dietary diversity among children (6-23 months)? Evidence from JEEViKA in Rural Bihar, India. PLoS ONE. (2023) 18:e0279724. 10.1371/journal.pone.0279724 36602987 PMC9815627

[65] Noack A-L Buggineni P Purty A Shalini S. Lessons from a Decade of Rural Transformation in Bihar. JEEViKA Learning Note Series, No. 8. (2021) 1–78. Available at: https://documents1.worldbank.org/curated/pt/298391515516507115/122290272_201800120100025/additional/122548-WP-P090764-PUBLIC-India-BRLP-Booklet-p.pdf (accessed February 7, 2024).

[66] Project Concern International. Quality Assessment and Support Process and Methodology. Available at: www.projectconcernindia.org (accessed January 14, 2024).

[67] Barkat A Ahamed FM Rabby MF Osman A Bedi A. Human Development Research Centre, and International Institute of Social Studies, Erasmus University of Rotterdam (ISS-EUR). Report on Annual Outcome Monitoring (AOM) 2022 of National Urban Poverty Reduction Programme [Report]. Dhaka (2022).

[68] Rajiv Gandhi Mahila Vikas Pariyojana Centre for Operations Research and Training and Centre for Operations Research and Training (CORT) Vadodara. Maternal Health Campaign in Uttar Pradesh - Key Findings from the Baseline/Endline Survey: An Overview - Draft Report. Vadodara: Centre for Operations Research and Training (CORT) (2019).

[69] Population Council. JEEViKA/PCI as an Exemplar platform for improving MIYCN. New Delhi (2018).

[70] Ministry Ministry of Rural Development Govt. of India. Aajeevika. Available at: https://aajeevika.gov.in/what-we-do/social-inclusion-and-social-development (accessed May 10, 2024).

[71] Rana MM Huan NV Thach NN Bach TX Cuong NT. Effectiveness of community-based infant and young child (IYCF) support group model in reducing child undernutrition among ethnic minorities in Vietnam (p. ii). (2017). Available at: https://www.ennonline.net/fex/58/communitysupportgroupvietnam (accessed February 7, 2024).

[72] Decision1719/QD-TTg Decision1719/QD-TTg of 2021 approving the National Target Program for socio-economic development in ethnic minority and mountainous areas for the period 2021-2030 phase I: from 2021 to 2025 issued by the Prime Minister. Hanoi.

[73] Vietnam News. National target programme on socio-economic development in ethnic minority areas approved. (2021). Available at: https://vietnamnews.vn/society/1059976/national-target-programme-on-socio-economic-development-in-ethnic-minority-areas-approved.html (accessed March 6, 2024).

[74] Wubshet L. Community-led development: perspectives and approaches of four member organizations. London: Qeios.

[75] USAID. Committed to change: USAID localization report FY 2023. (2023). Available at: https://www.usaid.gov/localization/progressreport/full-report-fy2023 (accessed October 15, 2024).

[76] Ingram G. Locally driven development: overcoming the obstacles. Brookings Global Working Papers #173. Cantre for Sustainable Development at Brookings. (2022). Available at: https://www.brookings.edu/wp-content/uploads/2022/05/Locally-Driven-Development.pdf (accessed October 15, 2024).

[77] Traverso-Yepez M Maddalena V Bavington W Donovan C. Community capacity building for health. SAGE Open. (2012) 2:215824401244699. 10.1177/2158244012446996

[78] Buckner L Carter H Ahankari A Banerjee R Bhar S Bhat S . Three-year review of a capacity building pilot for a sustainable regional network on food, nutrition and health systems education in India. BMJ Nutr Prev Health. (2021) 4:59–68. 10.1136/bmjnph-2020-000180 34308113 PMC8258077

[79] Watson D Mushamiri P Beeri P Rouamba T Jenner S Proebstl S . Behaviour change interventions improve maternal and child nutrition in sub-Saharan Africa: a systematic review. PLOS Glob Public Health. (2023) 3:e0000401. 10.1371/journal.pgph.0000401 36996036 PMC10062616

[80] Lamstein S Stillman T Koniz-Booher P Aakesson A Collaiezzi B Williams T . Evidence of Effective Approaches to Social and Behavior Change Communication for Preventing and Reducing Stunting and Anemia: Report from a Systematic Literature Review. Arlington, VA: USAID/Strengthening Partnerships, Results, and Innovations in Nutrition Globally (SPRING) Project (2014).

[81] Shahan Asif M Jahan F. Opening the policy space: the dynamics of nutrition policy making in Bangladesh. Montpellier: Agropolis International, Global Support Facility for the National Information Platforms for Nutrition initiative (2017).

[82] Resnick D Anigo KM Anjorin O Deshpande S. Voice, access, and ownership: enabling environments for nutrition advocacy in India and Nigeria. Food Sec. (2024) 16:637–58. 10.1007/s12571-024-01451-2 38770157 PMC11102356

[83] RESULTS UK. Concern Worldwide and University of Westminster. What Works for nutrition? Stories of success from Vietnam, Uganda and Kenya. Westminster (2015).

